# Comparison of non-invasive methods of assessing liver fibrosis in combination ART-experienced Zimbabweans

**DOI:** 10.4102/sajhivmed.v20i1.844

**Published:** 2019-04-11

**Authors:** Brenda Nherera, Kudakwashe Mhandire, Tinashe K. Nyazika, Alfred Makura, Cuthbert Musarurwa, Prichard T. Mapondera, Babill Stray-Pedersen, Hilda T. Matarira

**Affiliations:** 1 Department of Chemical Pathology, Faculty of Medicine, College of Health Sciences, University of Zimbabwe, Harare, Zimbabwe; 2 Letten Foundation Research House, Harare, Zimbabwe; 3 Malawi-Liverpool-Wellcome Trust Clinical Research Programme, University of Malawi College of Medicine, Blantyre, Malawi; 4 Division of Community Health, Stellenbosch University, Cape Town, South Africa; 5 Division of Women and Children, Institute of Clinical Medicine, University of Oslo, Oslo, Norway

## Abstract

**Background:**

The prevalence of morbidity and mortality associated with liver disease among HIV-infected individuals on combination antiretroviral therapy (ART) is high. Early screening of liver disease is essential, as it provides an opportunity for successful treatment. Hence, there is a need for reliable, inexpensive and non-invasive early markers of hepatic damage.

**Objectives:**

Non-invasive algorithms are available for assessing the extent of liver fibrosis as markers of ongoing inflammatory damage. This study compared the use of the FibroTest, Fibrosis-4 (FIB-4) index, APRI test and AST:ALT ratio in assessing liver fibrosis in combination ART-experienced individuals.

**Methods:**

In a comparative cross-sectional study, 79 participants between the ages of 8 and 62 years were recruited. The performance of each fibrosis algorithm was determined using established cut-off scores for clinically significant liver fibrosis.

**Results:**

The prevalence of liver fibrosis as determined by the FibroTest, FIB-4 index, APRI test and AST: ALT ratio were 19.0%, 21.5%, 12.7% and 79.7%, respectively. For individual biomarkers, A-2M concentration (*p* < 0.001) and AST activity (*p* = 0.003) remained significantly elevated in participants with fibrosis than those without as defined by FibroTest and APRI test, respectively, after adjustments for multiple comparisons.

**Conclusion:**

Our data demonstrate a high prevalence of asymptomatic liver fibrosis among combination ART-experienced individuals in Zimbabwe, and this warrants adequate monitoring of liver fibrosis in individuals on ART. Discordance of fibrosis results among the algorithms and individual biomarkers and calls for further work in identifying optimal biomarkers for detection of asymptomatic fibrosis.

**Keywords:**

Liver fibrosis; Non-invasive methods; Biomarkers; Combination anti-retroviral therapy; Zimbabwe.

## Introduction

Sub-Saharan Africa faces a substantial burden of liver disease, with mortality owing to cirrhosis doubling over the past three decades.^1^ Globally, liver disease accounts for 14% – 18% of deaths among the human immunodeficiency virus (HIV)-infected population and approximately 50% of deaths among hospitalised HIV-infected individuals.^2,3^ Morbidity and mortality associated with liver disease in sub-Saharan Africa remains higher among the HIV-infected population than in the uninfected population despite increasing access to combination antiretroviral therapy (ART).^3,4,5^ Longevity related to combination antiretroviral therapy among the HIV-infected population allows for the development of non-acquired immune deficiency syndrome (AIDS) events, such as nephrotoxicity, cardiovascular disease and liver complications, associated with chronic ART exposure, HIV infection itself and other comorbidities such as chronic hepatitis B virus (HBV) infection to a greater extent and hepatitis C virus (HCV) infection to a lesser axtent.^5,6^

Whilst the liver is a regenerative organ that is capable of complete resolution with early detection and treatment,^6,7^ patients with established cirrhosis or hepatocellular carcinoma (HCC) usually present late.^1^ Thus, timely diagnosis is critical for successful treatment, especially among HIV-infected individuals, hence the need for simple, accessible, accurate, point-of-care diagnostic technologies.^1,6,7^ In resource-limited settings, early detection of liver fibrosis is limited by the unavailability of routine, non-invasive and affordable screening methods. Liver fibrosis gives rise to liver disease owing to the excessive deposition of extracellular matrix (ECM) on the hepatocytes.^8^ The process is gradual and not life-threatening until very late stages of the disease.

The invasive liver biopsy (LB) is the gold standard method for assessing liver fibrosis but lacks a standard interpretation protocol. This makes interpretation of results subjective and often inaccurate.^9,10,11^ Furthermore, liver histology gives a limited picture of only that portion of the liver from which the biopsy is derived. In contrast, the less invasive serum or plasma biomarkers of liver function, such as enzymes, provide a preview of the entire status of the liver.^12,13^ Consequently, serum biomarkers are thought to offer a better alternative, as there are simple tests that are readily available, reproducible, easy to apply and, if well validated, can be effectively used to follow up and monitor patients.^9,14^ However, individual biomarkers of liver damage are limited predictors of hepatic fibrosis,^15,16^ hence mathematical models that combine routinely available individual biomarkers and patient parameters into scores have been developed to improve the sensitivity and specificity of detection of fibrosis.^8^ These models form algorithms such as the FibroTest, Fibrosis-4 (FIB-4) index, aspartate aminotransferase to alanine aminotransferase (AST: ALT) ratio and aspartate aminotransferase to platelet ratio index (APRI) test.^8,17^

FibroTest is a patented, non-invasive algorithm that has been shown to accurately predict liver fibrosis in different aetiologies of liver disease.^10,18^ FibroTest results have been shown to be comparable to the LB findings and have been used as an alternative diagnostic method in predicting liver fibrosis in several countries since 2002.^19,20^ Not so much the other algorithms despite their potential to improve access to diagnosis of liver fibrosis. Furthermore, these algorithms have not been validated in non-Caucasian populations, and their utility in African populations is sparsely reported in literature.

Transient elastography also referred to as the Fibroscan is another non-invasive, highly acceptable, rapid and painless method of assessing liver fibrosis.^21,22^ The technique is not serum or plasma based, but it uses both ultrasound and low-frequency elastic waves to quantify liver fibrosis. The method has been validated for liver fibrosis staging in patients with chronic liver diseases.^21^ The method is increasingly used in Europe; however, there are limited data on its utility in African populations.^22^ The present study aimed to determine the prevalence of significant liver fibrosis in ART-experienced individuals using four serum-based algorithms and comparing their performance.

## Materials and methods

### Study participants and sample collection

Between June and September 2014, we prospectively recruited 79 consecutive individuals from Harare Central Hospital and Parirenyatwa Group of Hospitals Opportunistic Infections Clinic, Harare, Zimbabwe. All our participants were HIV-infected and on ART for at least six months. Non-ambulatory patients, tuberculosis co-infected patients and pregnant women were excluded from the study. On enrolment, a questionnaire was administered to obtain medico-demographic data from each participant. Five millilitres (mL) of blood were collected from each participant into plain tubes and samples were centrifuged and had serum aliquoted within 2 h of bleeding.

One aliquot of serum was immediately analysed for ALT, AST, g-glutamyl transferase (GGT), total bilirubin (TBil) and HBV, whilst another aliquot was immediately frozen and kept at −80 °C for six weeks before measurement of haptoglobin, apolipoprotein A-1 (Apo A-1) and alpha-2 macroglobulin (A-2M) concentrations. Another 5 mL of blood were collected in ethylenediaminetetraacetic acid (EDTA) tubes and analysed for platelet count within 3 h of phlebotomy.

### Laboratory analysis

Platelet count was determined using a Sysmex XT-4000i automated Hematology analyser (Sysmex Corporation, Kobe, Japan). Haptoglobin, Apo A-1 and A-2M concen-trations were determined using the sandwich enzyme-linked immunosorbent assay (ELISA) method (Elabscience Biotechnology Co., Ltd, Wuhan, China). The serum activities of ALT, AST, GGT and TBil concentration were determined using the Beckman Coulter AU680 Chemistry analyser (Beckman Coulter, Inc., Mishima, Japan). Hepatitis B virus status was determined using the Hightop one step rapid HBV (5-in-1) test kit (Qingdao Hightop Biotech Co., Ltd, Shandong, China). All assays were performed following the manufacturer’s instructions.

### Algorithms for detection of fibrosis

The specific formulae used to determine the algorithm scores are shown in Table 1.

**TABLE 1:** Formulae of non-invasive algorithms for detection of fibrosis used in the study.

Formula	Equation
FibroTest	4.467 × log[A-2M (g/L)] – 1.357 × log[Haptoglobin (g/L)] + 1.017 × log[GGT (IU/L)] + 0.0281 × [Age (years)] + 1.737 × log[TBil (µmol/L)] – 1.184 × [Apo A-1 (g/L)] + 0.301 × Gender (Female = 0, Male = 1) – 5.540 (www.biopredictive.com)
FIB-4 index	[Age (years) × AST (IU/L)]/[Platelets (× 10^9^/L) × √ALT (IU/L)]
APRI test	[(AST (IU/L)/ ULN)/Platelet count (× 10^9^/ L)] × 100
AST: ALT ratio	AST/ALT

ULN, upper limit of normal, ULN of AST: 42 IU/L (according to the local laboratory standards); APRI, aminotransferase to platelet ratio index; FIB-4, Fibrosis-4 index; AST, aspartate aminotransferase; ALT, alanine aminotransferase.

### Statistical analysis

The Mann-Whitney test was used to compare non-parametric continuous variables between participants with significant fibrosis and those without. Kappa test was used to assess degree of agreement between algorithms. Cut-off values for significant hepatic fibrosis were as follows: APRI test > 0.5, FIB-4 index ≥ 1.45, FibroTest ≥ 0.32 and AST:ALT ratio > 1. These values have been reported to be predictive of significant hepatic fibrosis and were adopted for this study.^10,23,24,25^ All statistical analyses were performed using Stata 13.0 (Stata Corp., College Station, Texas, USA) software package and a *p*-value of < 0.05 was considered statistically significant.

## Ethical consideration

The study protocol was approved by the Joint Parirenyatwa Hospital and College of Health Sciences Research Ethics Committee (JREC Ref: 45/14). All participants gave written informed patient consent or assent. Consent was granted by parents or guardians in the case of minors.

## Results

### Characteristics of the study population

We enrolled 79 HIV-infected individuals with mean age and standard deviation (s.d.) of 41 and 11 years, respectively. The majority of participants (65.8%; *n* = 55) were female and the average body mass index (BMI) was 23 kg/m^2^, with 14.7% being underweight, 61.8% being normal weight, 14.7% being overweight and 8.8% being obese. Seventy-six per cent of the participants were on nucleoside reverse transcriptase inhibitor (NRTI) plus non-nucleoside reverse transcriptase inhibitors (NNRTI), 17.7% were taking NRTIs plus protease inhibitor (PI) whilst 6.3% were taking NRTIs only. The duration on ART ranged from 1 to 13 years with a median of four years and six months and interquartile range (IQR) of 2–7 years. Based on serological tests, 3.8% (*n* = 3) of study participants had HIV/HBV co-infection. The demographic characteristics of the study participants are shown in Table 2.

**TABLE 2:** Demographic characteristics of study participants.

Parameter	Participants (*n* = 79)
Gender: Females *n* (%)	52 (65.8)
Age (years) mean ± s.d.	41 ± 11
Height (metres) median (IQR)	1.70 (1.60–1.70)
Weight (kg) median (IQR)	66 (56–75)
BMI (kg/m^2^) mean ± s.d.	23 ± 4.4
CD4+ count (cells/uL) median (IQR)	416 (254–624)
HIV/HBV co-infection, *n* (%)	3 (3.8)
Period on ART (years) median (IQR)	4.5 (2–7)
Patients taking alcohol, *n* (%)	11 (13.9)

s.d., standard deviation; IQR, interquartile range; BMI, body mass index; HIV/HBV, human immunodeficiency virus/hepatitis B virus; ART, antiretroviral therapy.

### Utility of algorithms for the prediction of hepatic fibrosis

**FIGURE 1:**
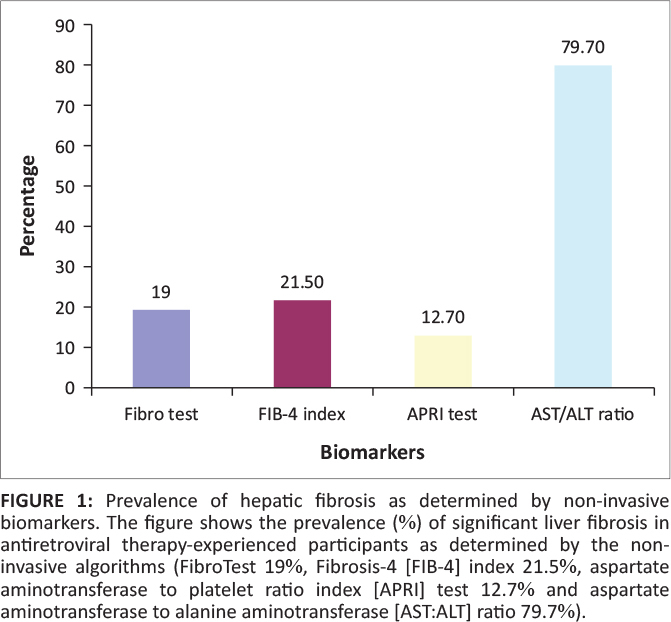
Prevalence of hepatic fibrosis as determined by non-invasive biomarkers. The figure shows the prevalence (%) of significant liver fibrosis in antiretroviral therapy-experienced participants as determined by the non-invasive algorithms (FibroTest 19%, Fibrosis-4 [FIB-4] index 21.5%, aspartate aminotransferase to platelet ratio index [APRI] test 12.7% and aspartate aminotransferase to alanine aminotransferase [AST:ALT] ratio 79.7%).

We first determined the prevalence of fibrosis in our study participants using each of the four algorithms (FibroTest, FIB-4 index, APRI test and AST:ALT ratio). The prevalence of fibrosis according to each algorithm were: FibroTest (19%), FIB-4 index (21.5%), APRI test (12.7%) and AST:ALT ratio (79.7%), as shown in Figure 1.

The average prevalence of fibrosis was 17.7% using the three comparable algorithms (FibroTest, FIB-4 index and APRI test) but increased to 33.2% when AST:ALT ratio was included. Notably, 19.4% (*n* = 7) of the 36/79 (45.6%) participants receiving nevirapine-containing ART regimens had significant fibrosis based on the FibroTest, which has been validated in other settings.^19,20^ One of the three participants co-infected with HBV had significant fibrosis as determined by the FibroTest. When we performed the test for agreement among the non-invasive algorithms, there was a moderate agreement between FIB-4 index and APRI test (*k* = 0.46), fair agreement between (1) FibroTest and FIB-4 index (*k* = 0.40) and (2) between FibroTest and APRI test (*k* = 0.25). The AST:ALT ratio was in poor agreement with all three other algorithms: FibroTest (*k* = 0.08), FIB-4 index (*k* = 0.10) and APRI test (*k* = 0.08).

### Individual biomarkers in fibrosis and non-fibrosis as defined by FibroTest

We compared individual serum biomarkers between participants with significant fibrosis and those without as defined by FibroTest. Total bilirubin and A-2M concentrations were significantly elevated in participants with fibrosis, median (IQR) 7 μmol/L (5–46) versus 5 μmol/L (4–7) (*p* = 0.029) and 1.5 g/L (1.1–2.9) versus 0.2 g/L (0.1–0.8) (*p* < 0.001), respectively, when compared to those without fibrosis. However, after adjusting for multiple comparisons with the Bonferroni adjustment, only A-2M (*p* < 0.001) remained significant. The findings are summarised in Table 3.

**TABLE 3:** Comparison of biomarkers in participants with significant fibrosis and those without as defined by the FibroTest.

Variable	Participants with no fibrosis (*n* = 63)		Participants with fibrosis (*n* = 16)		*p*
	Median	IQR	Median	IQR	
AST (IU/L)	28	24–37	33	27–39	0.366
ALT (IU/L)	24	16–36	22	16–31	0.696
GGT (IU/L)	39	25–59	51	29–75	0.566
TBil (µmol/L)	5	4–7	7	5–46	**0.029** 0.203*
Apo A-1 (g/L)	0.94	0.53–1.58	1.38	0.47–1.66	0.579
Haptoglobin (g/L)	0.037	0.012–0.064	0.019	0.003–0.065	0.542
A-2M (g/L)	0.196	0.092–0.811	1.501	1.065–2.893	< 0.001 < 0.001*

TBil, total bilirubin; GGT, g-glutamyl transferase; AST, aspartate aminotransferase; ALT, alanine aminotransferase; Apo A-1, apolipoprotein A-1; A-2M, alpha-2 macroglobulin; IQR, interquartile range; IU/L.

For all statistical analyses and bold *p*-values in tables, significance was set at 0.05.

* , Bonferroni adjusted *p*-value.

### Individual biomarkers in fibrosis and non-fibrosis defined by aspartate aminotransferase to platelet ratio index test

We further compared individual serum biomarkers based on APRI test strata. Aspartate aminotransferase and Apo A-1 were significantly elevated in participants with fibrosis median (IQR) 50 (32–77) IU/L versus 28 (23–36) IU/L (*p* = 0.005) and 1.6 (1.2–1.8) g/L versus 0.9 (0.5–1.5) g/L (*p* = 0.027), respectively, when compared to those without and after adjusting for multiple comparisons with the Bonferroni adjustment, only AST (*p* = 0.003) remained significant. Table 4 summarises these findings.

**TABLE 4:** Comparison of biomarkers in participants with significant fibrosis and those without as defined by the aspartate aminotransferase to platelet ratio index test.

Variable	Participants with no fibrosis (*n* = 68)		Participants with fibrosis (*n* = 11)		*p*
	Median	IQR	Median	IQR	
AST (IU/L)	28	23–36	50	32–77	**0.005** 0.003*
ALT (IU/L)	22	16–32	31	19–73	0.151
GGT (IU/L)	38	25–61	53	36–102	0.266
TBil (umol/L)	5	4–7	4	4–7	0.371
Apo A-1 (g/L)	0.926	0.477–1.521	1.585	1.215–1.814	**0.027** 0.186*
Haptoglobin (g/L)	0.037	0.008–0.059	0.039	0.013–0.101	0.681
A-2M (g/L)	0.310	0.114–0.974	0.663	0.097–1.430	0.723

AST, aspartate aminotransferase; ALT, alanine aminotransferase; GGT, g-glutamyl transferase; TBil, total bilirubin; Apo A-1, apolipoprotein A-1; A-2M, alpha-2 macroglobulin; IQR, interquartile range; IU/L.

For all statistical analyses and bold *p*-values in tables, significance was set at 0.05.

* , Bonferroni adjusted *p*-value.

### Correlation between significant fibrosis and participants’ characteristics

A correlation between significant fibrosis according to FIB-4 index and patients’ characteristics, which were age, gender, BMI, CD4+ cell count and period on ART, was performed. Only age correlated significantly (*p* = 0.0058), suggesting there is an association between old age and the presence of hepatic fibrosis.

## Discussion

Data on the epidemiology and prevalence of liver disease are essential for the awareness, diagnosis, management and prioritisation of public health resources.^1^ In this study, we observed a moderately high prevalence of asymptomatic liver fibrosis (12.7% – 21.5%) based on FibroTest, APRI test and FIB-4 index. This, to our knowledge, is the first data from a Zimbabwean population to demonstrate liver fibrosis in ART-experienced patients using the algorithms. Our observed prevalence of liver fibrosis was higher when compared to other studies that have quantified the presence of hepatic fibrosis in HIV-infected individuals using different non-invasive serum algorithms.

A number of factors are said to contribute to the development of liver fibrosis in HIV-infected patients. The virus itself has been shown to cause liver fibrosis by activating hepatic stellate cells, which are the principal fibrogenic cells in the liver.^5,6,26^ Liver fibrosis in this group of patients can be drug-induced. Some studies have shown that nevirapine-containing regimens are associated with an increased risk of liver fibrosis because nevirapine causes direct hepatic damage.^2^ Our study demonstrated that 19.4% of the 45.6% of participants receiving nevirapine-containing regiments had significant fibrosis. Other risk factors associated with the development of fibrosis in this group of patients include cardiovascular diseases, diabetes mellitus, dyslipidaemias, being obese and ageing.^26^

Human immunodeficiency virus-positive individuals usually present with thrombocytopenia^27^ and the APRI test makes use of platelet count in its formula; this in turn falsely increases the prevalence of significant fibrosis as determined by the APRI test. In a Kenyan study, the APRI test was performed on HIV-monoinfected patients and they obtained a prevalence of 8.6%; a study done in the US obtained a prevalence of 8.3% and another study called the Strategic Timing of Anti-Retroviral Treatment (START) trial with a hetero-geneous population of Asians, Europeans and Australians obtained a prevalence of 8.5%.^6,28,29^ These prevalences were all lower than the 12.7% that we obtained in this study. The US study used a cut-off of > 1.5, whilst our study, the Kenyan study, and the START trial used a cut-off of > 0.5, and this could have lowered the prevalence of fibrosis in the US study.

A Moroccan study that performed the FIB-4 index on HIV-monoinfected participants observed a prevalence of 15.5%, which was higher than the 10% obtained by the START trial.^6,30^ Our study found a prevalence of 21.5%, which was higher than the prevalence in both Morocco and the START trial. Our mean age was higher (41 years) compared to the Moroccan (39.8 years) and the START trial (35 years), which could have consequently increased our prevalence as the FIB-4 index incorporates age in its formula and age, has been found to be a risk factor for development of fibrosis.^28,31^

The kappa analysis we performed demonstrated that the FIB-4 index, APRI test and FibroTest performed comparably. Concordance between FIB4-index and FibroTest has been reported elsewhere in a study conducted on individuals with HCV monoinfection (*k* = 0.561).^25^ A moderate agreement between the APRI test and FIB-4 index has also been shown in an HIV monoinfection population (*k* = 0.573)^32^ and in an HCV monoinfection population (*k* = 0.507),^33^ and these results are comparable to the *k* = 0.46 that we observed in our study. Another study compared the APRI test and the FIB-4 index to the LB in patients with non-alcoholic fatty liver disease and obtained a fair and statistically significant agreement, APRI test (*k* = 0.33) and FIB-4 index (*k* = 0.34).^34^ These results further confirm that the two tests are comparable in different liver fibrosis aetiologies and even against the LB, which is the gold standard test. In contrast, AST:ALT ratio performance was not comparable to any of the other three algorithms, showing poor agreement with any of the three algorithms. Therefore, we speculate that this discordance could be owing to overexpression of AST from non-hepatic sources and also delayed clearance of AST relative to ALT.^35,36^ Thus, making AST-based algorithms problematic in assessing liver fibrosis by overestimating the degree of liver fibrosis. AST:ALT ratio consequently becomes a very unreliable test for estimating significant fibrosis in ART-experienced individuals. Among the comparable algorithms, FIB-4 index is the best in terms of reliability and ease of performing because it includes age in its calculations and the tests involved in calculation are routinely available.

We compared individual biomarkers between fibrotic and non-fibrotic individuals as defined by the FibroTest and APRI test to determine the possible utility of using a single biomarker in prediction of fibrosis. Only AST and A2M remained significantly higher in patients with liver fibrosis compared to those without, adjusting for multiple comparisons. We therefore speculate that the observed significance could be because of the fact that (1) most of the AST in hepatocytes is located in the mitochondria and damage to hepatocytes causes the release of both the cytoplasmic and mitochondrial AST, leading to a raised AST in liver fibrosis,^35^ and (2) alpha-2 macroglobulin, a PI, is produced by hepatocytes, granuloma cells and hepatic stellate cells during inflammation. Hence, its synthesis is increased as the body tries to inhibit breakdown of ECM proteins, which favours fibrosis.^37^ In our study, A-2M was able to strongly distinguish fibrosis from non-fibrosis and this has also been demonstrated in a study conducted in Romania.^37^ Aspartate aminotransferase significantly distinguished fibrosis from non-fibrosis, but the enzyme is not a good individual biomarker of liver fibrosis as it is not solely produced by the liver and this results in false-positives. On the contrary, A-2M is an excellent individual biomarker of liver fibrosis, but analysis of this protein is costly, thus making it not ideal for routine assessment of liver fibrosis in poor resource settings.

This study was limited by the unavailability of liver LB, which is the gold standard method for assessing liver fibrosis. We could not assay LB on our patients as it is ethically unacceptable for routine monitoring of liver fibrosis, particularly in the HIV-infected individuals because it increases the risks of coagulopathies.^8,35,38^ However, including LB in our study could have potentially helped us in drawing a stronger conclusion on the diagnostic performance of each algorithm. Transient elastography was going to be a better comparator among the non-invasive tools of assessing liver fibrosis. However, the test was not performed owing to a lack of availability of the test and also budget constraints. Cut-off values used in our study were based on studies performed mainly in Europe, with most participants having HCV.^18,39^ Although we have no reason to believe that the cut-off values would differ in our setting, studies to validate the cut-off values in an African population are recommended.

In conclusion, this is the first study in Zimbabwe to demonstrate that algorithms such as FIB4-index, APRI test and FibroTest together with individual serum biomarkers like A-2M can be used as alternative methods for assessing liver fibrosis in asymptomatic, HIV-infected individuals on ART. The moderately high prevalence of asymptomatic liver fibrosis obtained in this study warrants adequate monitoring among ART-experienced individuals. The discordance of fibrosis results among the algorithms, and individual biomarkers call for further work in identifying optimal biomarkers for detection of asymptomatic fibrosis. However, AST:ALT ratio does not require further work, as it has been shown to be an unreliable test for assessing liver fibrosis. Whilst a number of studies, including the Ugandan study,^16^ have demonstrated high levels of fibrosis in HIV-monoinfected patients, the natural history and long-term liver outcomes in this group of patients have not been well described. There is, therefore, an urgent need to have well-designed cohort studies looking at long-term outcomes in this group of patients and an easy to apply non-invasive test in this setting. Introducing better non-invasive markers of liver fibrosis in Zimbabwe and sub-Saharan Africa has a potential of simplifying and improving the way ART-experienced patients are monitored for liver fibrosis.
